# Comprehensive Assessment of the Impact of Blood Pressure, Body Mass Index, Smoking, and Diabetes on Healthy Life Expectancy in Japan: NIPPON DATA90

**DOI:** 10.2188/jea.JE20240298

**Published:** 2025-08-05

**Authors:** Rumi Tsukinoki, Yoshitaka Murakami, Takehito Hayakawa, Aya Kadota, Akiko Harada, Yoshikuni Kita, Akira Okayama, Katsuyuki Miura, Tomonori Okamura, Hirotsugu Ueshima

**Affiliations:** 1 Department of Public Health Nursing, Institute of Science Tokyo, Tokyo, Japan; 2 Department of Clinical Nursing, Shiga University of Medical Science, Otsu, Japan; 3 Department of Medical Statistics, Faculty of Medicine, Toho University, Tokyo, Japan; 4 The Kinugasa Research Organization, Ritsumeikan University, Kyoto, Japan; 5 NCD Epidemiology Research Center, Shiga University of Medical Science, Otsu, Japan; 6 Department of Public Health, Shiga University of Medical Science, Otsu, Japan; 7 Department of Medical Statistics, Shiga University of Medical Science, Otsu, Japan; 8 Faculty of Nursing Science, Tsuruga Nursing University, Fukui, Japan; 9 Research Institute of Strategy for Prevention, Tokyo, Japan; 10 Department of Preventive Medicine and Public Health, Keio University, Tokyo, Japan

**Keywords:** healthy life expectancy, blood pressure, body mass index, smoking, diabetes, nationwide cohort study

## Abstract

**Background:**

Healthy life expectancy (HLE) is a population health indicator that is widely used in developed countries, but little is known about its relationships with combinations of non-communicable disease risk factors. This study was conducted to examine HLE at age 65 years according to combinations of blood pressure levels, body mass index, smoking status, and diabetes mellitus (DM) in a Japanese population.

**Methods:**

In a nationwide cohort study (NIPPON DATA90), data on these risk factors were obtained from participants in 1990 through physical examinations, blood tests, interviews, and questionnaires. Subsequently, participants aged ≥65 years underwent surveys on activities of daily living in 1995 and 2000, and multistate life tables were used to calculate combination-specific HLEs and their 95% confidence intervals (CIs).

**Results:**

The study population comprised 6,569 participants (men: 2,797; women: 3,772) who were followed until 2010. HLE at age 65 years in men with grade II/III hypertension, obesity, current smoker status, and DM (HLE 12.9; 95% CI, 12.9–13.0 years) was 9.7 years shorter than men without these risk factors (HLE 22.6; 95% CI, 22.4–22.8 years). Similarly, HLE at age 65 years in women with grade II/III hypertension, obesity, current smoker status, and DM (HLE 16.2; 95% CI, 15.9–16.5 years) was 10.1 years shorter than women without these risk factors (HLE 26.3; 95% CI, 26.3–26.3 years).

**Conclusion:**

The large discrepancies in HLEs underscore the impact of non-communicable disease risk factors, which should be considered when formulating health interventions to improve HLE in Japanese older adults.

## INTRODUCTION

Healthy life expectancy (HLE) is a composite health measure that incorporates information on mortality and morbidity to estimate the remaining number of years a person is expected to live in good health.^1^ HLE has become a common indicator of population health in developed countries that already have long life expectancies.^2^ In 2020, the United States government included HLE in its “Healthy People 2030” program as a leading health indicator to monitor the nation’s health and well-being.^3^

Non-communicable diseases (NCDs) are major determinants of HLE. For example, hypertension, obesity, smoking, and diabetes mellitus (DM) are known NCD risk factors that can impact the length and quality of life.^2^^,^^4^^,^^5^ Previous studies from the United States^6^^–^^9^ and Europe^7^^,^^9^^,^^10^ have reported that people with NCD-related lifestyle risk factors have shorter HLEs than those without these factors. Those studies examined the simultaneous effects of multiple risk factors through the use of combined risk scores calculated based on the total number of factors present in an individual. Although combined risk scores are useful for indicating the overall relationships between risk factors and HLE,^6^^,^^7^^,^^10^ they do not provide insight into each factor’s relative influence on HLE.

An analysis of three cohorts in Denmark, Germany, and Norway utilized multistate Markov models to calculate the independent and joint effects of smoking, physical activity, alcohol consumption, and obesity on life expectancy with and without cardiovascular disease (CVD).^9^ Generating different combinations of risk factors and measuring the HLEs of each combination provides an alternative approach to the use of combined risk scores. Comparing such combination-specific HLEs in detail may be a more precise way to explore and identify major risk factors for HLE. Accordingly, this approach could provide valuable information when formulating strategies for NCD risk factor management in health care and public health interventions. However, no studies on HLEs based on combinations of NCD risk factors have been conducted in the Asia-Pacific region.

In this Japanese nationwide cohort study, we examined HLEs at age 65 years according to combinations of common NCD risk factors, including blood pressure (BP) levels, body mass index (BMI), smoking status, and DM. Multistate life tables were used to calculate the combination-specific HLEs of participants.

## METHODS

### Study design and study population

The National Integrated Project for Prospective Observation of Non-communicable Disease and its Trends in the Aged, 1990 (NIPPON DATA90) was a nationwide cohort study conducted on 8,383 community residents (men: 3,503; women: 4,880) aged ≥30 years from 300 randomly selected areas throughout Japan.^11^^–^^15^ In NIPPON DATA90, study participants were all community-dwelling people who could come and participate in a health examination by themself. No people from medical institutions or nursing homes participated in this examination. A multistage sampling method was employed to avoid sampling bias during the participant selection process. In 1990, participants underwent a baseline examination consisting of a physical examination, blood tests, structured interview, and self-administered medical history and lifestyle questionnaires. The participants were subsequently followed from 1990 until November 15, 2010. NIPPON DATA90 conducted a two-wave survey of activities of daily living (ADL) for participants aged ≥65 years in 1995 and 2000. These ADL surveys were conducted through face-to-face interviews at home, telephone interviews, or questionnaires sent via mail. For this study, we focused on participants aged 40 to 90 years who had undergone the ADL surveys in 1990, 1995, or 2000. After excluding 1,709 participants who were aged under 40 years or 90 years and older, as well as 105 participants with missing data (BP, BMI, smoking status, DM, or ADL), the study population comprised 6,569 participants.

### Study outcome

The study outcome was HLE at age 65 years. We used the NIPPON DATA90 database to estimate HLE. The cause of death of NIPPON DATA90 was confirmed by the International Classification of Diseases Revisions 9 (ICD-9) and 10 (ICD-10) from the National Vital Statistics database of Japan. ADL was used as a morbidity index to calculate HLEs. The NIPPON DATA90 ADL surveys utilized the Katz ADL index,^16^ and participants answered five items from this index (feeding, dressing, bathing, toileting, and transferring/walking indoors). We defined ‘disabled’ participants in the study population using the Katz ADL index. This approach was routinely used to estimate healthy life expectancy in Japan.^17^^,^^18^ In these papers, the participants who met at least one of the five questions of the Katz ADL index were defined as ‘disabled.’

### Risk factors

We analyzed the following four NCDS risk factors that were determined during the NIPPON DATA90 baseline examination: BP levels, BMI, smoking status, and DM. BP levels were measured by trained observers using a standard mercury sphygmomanometer on the right arm with participants in a seated position. The use of antihypertensive agents was not considered because we sought to evaluate the effects of increased BP levels, which can also occur in hypertensive patients under treatment.^12^ BMI was calculated as weight (kg) divided by the square of height (m). Smoking status was ascertained through interviews with public health nurses. DM was identified through blood tests and medical history questionnaires. Blood samples were analyzed at a central laboratory (SRL Inc., Tokyo, Japan) using previously established methods.^11^^–^^15^ HbA1c levels were measured using Japan Diabetes Society units and converted to National Glycohemoglobin Standardization Program equivalent values using the formula: HbA1c (%; National Glycohemoglobin Standardization Program) = HbA1c (%; Japan Diabetes Society) + 0.4%.^19^^,^^20^

### Statistical analysis

We generated multiple combinations of the four risk factors and calculated the combination-specific HLEs using multistate life tables. First, continuous variables (BP and BMI) were converted into polytomous variables. BP was categorized into the following four groups in accordance with the Japanese Society of Hypertension’s 2019 guidelines^21^: (1) normal BP: systolic BP <120 mm Hg and diastolic BP <80 mm Hg, (2) high normal/elevated BP: systolic BP 120–139 mm Hg and/or diastolic BP 80–89 mm Hg, (3) grade I hypertension: systolic BP 140–159 mm Hg and/or diastolic BP 90–99 mm Hg, and (4) grade II/III hypertension: systolic BP ≥160 mm Hg and/or diastolic BP ≥100 mm Hg. These cut-off levels for BP are similar to those stipulated in the European Society of Cardiology/European Society of Hypertension guidelines.^22^ Next, BMI was categorized into the following four groups: (1) underweight: BMI <18.5 kg/m^2^, (2) normal weight: BMI 18.5–24.9 kg/m^2^, (3) overweight: BMI 25.0–29.9 kg/m^2^, and (4) obese: BMI ≥30.0 kg/m^2^. Smoking status was categorized into the following three groups: (1) never-smokers, (2) ex-smokers, and (3) current smokers. Participants with DM were identified as those with a history of DM, Hb1Ac ≥6.5%, and/or diabetes treatments. Based on BP (four categories), BMI (four categories), smoking status (three categories), and DM (two categories), we generated a total of 96 risk factor combinations (4 × 4 × 3 × 2) for analysis.

A multistate Markov transition model for disability and mortality was constructed (eFigure 1). The model consisted of two non-absorbing states (non-disabled and disabled, defined using the Katz ADL index) and one absorbing state (death). We set four possible health transitions over time: (1) moving from non-disabled to disabled (occurrence of a disabled state), (2) moving from disabled to non-disabled (recovery from a disabled state), (3) moving from non-disabled to death, and (4) moving from disabled to death. The probabilities for these four transitions were estimated using a multinomial logistic regression model (eTable 1).

We constructed multistate life tables to calculate the sex-specific HLEs at age 65 years and their 95% confidence intervals (CIs) for each of the 96 risk factor combinations. All estimations for the multistate life tables were derived using the Stochastic Population Analysis for Complex Events (SPACE) program.^23^ In SPACE, we set 1,000 bootstrap samples in simulation. We set the primary sampling unit as an identical number of individuals, which indicated an individual was treated as a stratified simple random sample. Analyses were performed using SAS Release 9.40 (SAS Institute, Cary, NC, USA).

## RESULTS

Table 1 summarizes the characteristics of the 6,569 participants (men: 2,797; women: 3,772) in the study population. The mean follow-up duration was 16.3 (standard deviation [SD], 5.7) years in men and 17.5 (SD, 5.0) years in women. The mean age at baseline was 57.5 (SD, 11.5) years in men and 57.5 (SD, 11.7) years in women. The prevalence of the risk factors was as follows: grade II/III hypertension (men: 22.0%; women: 17.8%), obesity (men: 1.6%; women: 3.3%), current smokers (men: 52.7%; women: 9.0%), and DM (men: 10.3%; women: 6.2%).

**Table 1.** Participant characteristics

	Men (*n* = 2,797)			Women (*n* = 3,772)		
	
	Number	Mean	SD	Number	Mean	SD
Follow-up duration (years)	2,797	16.3	(5.7)	3,772	17.5	(5.0)
Age at baseline (years)	2,797	57.5	(11.5)	3,772	57.5	(11.7)
Systolic BP (mm Hg)	2,797	140.4	(20.3)	3,772	137.8	(20.4)
BMI (kg/m^2^)	2,797	23.0	(3.0)	3,772	23.1	(3.3)

Risk factors	Number	%		Number	%	

BP^a^						
Normal	276	9.9		570	15.1	
High normal/elevated	1,014	36.3		1,364	36.2	
Grade I hypertension	892	31.9		1,165	30.9	
Grade II/III hypertension	615	22.0		673	17.8	
BMI^b^						
Underweight	175	6.3		231	6.1	
Normal weight	1,959	70.0		2,537	67.3	
Overweight	618	22.1		880	23.3	
Obese	45	1.6		124	3.3	
Smoking status						
Never-smoker	607	21.7		3,340	88.5	
Ex-smoker	716	25.6		91	2.4	
Current smoker	1,474	52.7		341	9.0	
Diabetes mellitus^c^						
Yes	287	10.3		233	6.2	
No	2,510	89.7		3,539	93.8	

BMI, body mass index; BP, blood pressure; SD, standard deviation.

^a^Normal BP: systolic BP <120 mm Hg and diastolic BP <80 mm Hg; High normal/elevated BP: systolic BP 120–139 mm Hg and/or diastolic BP 80–89 mm Hg; Grade I hypertension: systolic BP 140–159 mm Hg and/or diastolic BP 90–99 mm Hg; Grade II/III hypertension: systolic BP ≥160 mm Hg and/or diastolic BP ≥100 mm Hg.

^b^Underweight: BMI <18.5 kg/m^2^; Normal weight: BMI 18.5–24.9 kg/m^2^; Overweight: BMI 25.0–29.9 kg/m^2^; Obese: BMI ≥30.0 kg/m^2^.

^c^Diabetes mellitus was defined as a history of diabetes mellitus, Hb1Ac ≥6.5%, and/or use of antidiabetic medications.

**Figure 1.**
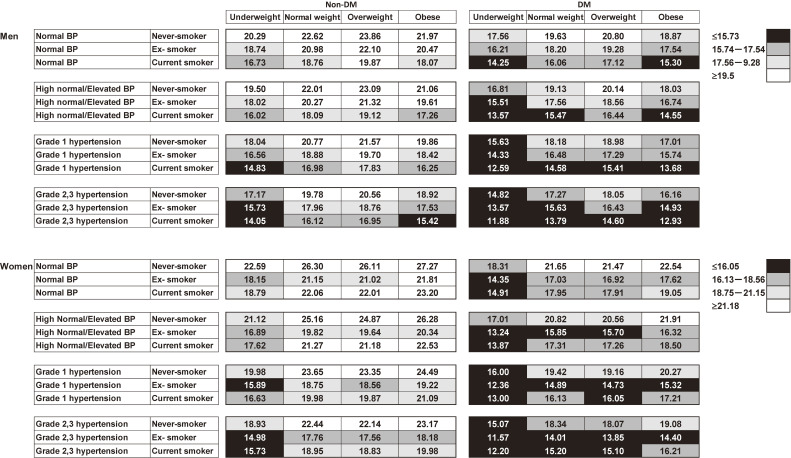
Sex-specific healthy life expectancies (years) at age 65 years according to combinations of blood pressure levels, body mass index, smoking status, and diabetes mellitus in a Japanese population. Healthy life expectancy was divided into quartiles for men and women, and the four categories were color-coded (men: ≤15.73, 15.74–17.54, 17.56–19.28, and ≥19.5 years; women: ≤16.05, 16.13–18.56, 18.75–21.15, and ≥21.18 years). Blood pressure (BP) was categorized into four groups (normal BP, high normal/elevated BP, grade I hypertension, and grade II/III hypertension). Body mass index (BMI) was categorized into four groups (underweight, normal weight, overweight, and obese). Smoking status was categorized into three groups (never-smokers, ex-smokers, and current smokers). Diabetes mellitus (DM) was defined as a history of DM, Hb1Ac ≥6.5%, and/or diabetes treatments.

The sex-specific HLEs at age 65 years according to the combinations of BP, BMI, smoking status, and DM are shown in Figure 1 and eTable 2. There was a difference of 9.7 years in HLE between high-risk men with grade II/III hypertension, obesity, current smoker status, and DM (12.9; 95% CI, 12.9–13.0 years) and low-risk men with normal BP, normal weight, never-smoker status, and no DM (22.6; 95% CI, 22.4–22.8 years). Similarly, there was a difference of 10.1 years in HLE between high-risk women with grade II/III hypertension, obesity, current smoker status, and DM (16.2; 95% CI, 15.9–16.5 years) and low-risk women with normal BP, normal weight, never-smoker status, and no DM (26.3; 95% CI, 26.3–26.3 years).

As an overall trend, HLEs at age 65 years in the no-DM groups were longer than those in the DM groups (men: 2.2–3.1 years; women: 3.4–4.7 years). In both the no-DM and DM groups, HLEs at age 65 years lineally decreased as BP levels increased. Next, HLEs at age 65 years in the grade II/III hypertension groups were generally shorter than those in the normal BP groups (men: 2.3–3.3 years; women: 3.4–4.7 years). For each category of BP, HLEs at age 65 years in the current smoker groups were found to be shorter than those in the never-smoker groups (men: 2.9–4.0 years; women: 2.9–4.1 years). Furthermore, we found that HLEs at age 65 years were shorter in men in the underweight groups (1.8–2.7 years) and obese groups (0.4–1.2 years) than those in the normal weight groups. However, HLEs at age 65 years were only shorter in women in the underweight groups (2.4–4.0 years) than those in the normal weight groups. These trends were mostly similar regardless of DM, BP, or smoking status.

## DISCUSSION

In this nationwide cohort study, we examined the relationships between HLEs at age 65 years and various combinations of NCD risk factors in a Japanese population. The analysis showed large differences in HLEs at age 65 years between the low-risk and high-risk groups in both men (9.7 years) and women (10.1 years). In addition, participants with combinations of multiple NCD risk factors (DM, hypertension, and smoking status) generally had lower HLEs than those without these factors. Among the individual risk factors, smoking status demonstrated the largest impact on HLE, followed by BP and DM. In contrast, being underweight or obese exerted only modest effects on HLE in our study population.

This comprehensive risk assessment is the first in the Asia-Pacific region to show the impact of common NCD risk factors on HLE in older adults. In Asia, rapid population aging and lifestyle changes have continued for the past 50 years.^24^ As a consequence, increasing HLE for older adults has become a crucial issue for public health policy. However, Asian people generally have different lifestyles from those of people in Europe and North America, which can manifest as different distributions of NCD risk factors.^22^ This may in turn give rise to diverse risk profiles that have discrepant effects on HLE. Furthermore, Japan currently has one of the longest HLEs in the world.^17^^,^^25^^,^^26^ Our findings may help to inform the management of NCD risk factors in Japan as well as other Asian countries.

Data from the United States Nurses’ Health Study and the Health Professionals Follow-Up Study indicated that adherence to four or five low-risk lifestyle factors (smoking status, physical activity, alcohol consumption, BMI, and diet) was associated with a longer life expectancy at age 50 years in both men (7.6 years) and women (10 years).^6^ Similarly, a multicohort study conducted in four European countries reported that smoking, physical inactivity, and obesity were predictors of lower HLE.^7^ Another study of three European cohorts showed that a favorable lifestyle (never-smoker, physically active, light/moderate drinker, and overweight but not obese) could prolong CVD-free life expectancy at age 50 years by 29.7–38.5 years in women and 25.5–31.6 years in men.^9^ In the Netherlands, the Rotterdam Study showed that persons with the highest lifestyle scores based on five lifestyle factors (smoking status, physical activity, alcohol consumption, weight status, and diet quality) had a longer life expectancy free of heart failure at age 65 years in both men (4.4 years) and women (3.1 years) compared with persons with the lowest lifestyle scores.^10^ All these studies used a “combined risk score” approach calculated from the total number of risk factors present in an individual. A drawback of that approach is that the contribution of each risk factor is not apparent. In contrast, this study focused on the impacts of blood pressure, body mass index, smoking, and diabetes on HLEs in Japan. All these risk factors were routinely measured and available from health examinations. Disseminating the HLEs of these risk factors to society will encourage people to improve their lifestyle and health by using health examination data. These figures can lead people to reduce blood pressure, maintain normal weight, and quit smoking.

In our study, the impact of being underweight or obese was weaker than that of the other NCD risk factors. This finding may have been influenced by two different reasons. First, the proportion of Japanese people who are obese is relatively low, which could have been reflected in its reduced impact on HLE. Second, lower BMI and CVD risk may be confounded by cigarette smoking. Previous studies have shown that current and ex-smokers tend to have lower BMI and higher mortality risk than never-smokers,^27^^,^^28^ which may have distorted the observed relationship between BMI and HLE.

Our study’s strengths include the use of a nationwide cohort study, in which participants of NIPPON DATA90 were selected as a nationally representative sample. In addition, our study data, such as HbA1c levels and BP levels, were precisely measured using standardized methods. In contrast, other HLE studies tended to rely on lifestyle risk factors ascertained through self-administered questionnaires.^6^^,^^7^^,^^9^^,^^10^ In this way, our results were based on a representative sample with precise measurements that minimize selection and information bias. However, our findings should be interpreted with consideration to several limitations. First, some groups of ex-smokers and underweight persons showed longer HLEs than corresponding groups of non-smokers and overweight persons. These seemingly paradoxical results may be due to reverse causality, in which participants developed health problems that subsequently led to smoking cessation or weight loss. Second, the risk factors were only measured in the baseline examination, and subsequent changes in these factors were not considered in our study. Therefore, we could not determine if there were changes in BP, BMI, smoking habits, or DM status during the follow-up period. Such misclassifications could lead to the underestimation of differences in HLEs among the groups, which would suggest that our results are conservative. Third, we could not account for advances in diagnosis and medical treatment during follow-up periods. Fourth, we explored major NCD risk factors in HLE estimation. Other risk factors might affect our results of HLE, and the interpretation of the results should be cautious with these residual confounders.

### Conclusions

In this analysis of the relationships between HLE at age 65 years and combinations of four common NCD risk factors in a Japanese population, we found large differences in HLEs between low-risk participants (without any of the risk factors) and high-risk participants (with all four risk factors). Among the individual risk factors, smoking had the largest impact on HLE, followed by BP, DM, and underweight/obesity. These findings may help to identify older adults at risk of having shorter HLEs and to support the development of efficient strategies for NCD risk factor management.

## SUPPLEMENTARY MATERIAL

eTable 1. Parameter estimates of logistic regression for estimating transition rates in multistate Markov transition model

eTable 2. The Healthy life expectancies at age 65 years according to BP, Smoking status, DM, and BMI using NIPPON DATA 90 (*n* = 6,676; 2,797 men and 3,772 women)

eFigure 1. Multistate Markov model used with individuals being in one of three possible states at time.
