# Supplementary material for: Comprehensive Assessment of the Impact of Blood Pressure, Body Mass Index, Smoking, and Diabetes on Healthy Life Expectancy in Japan: NIPPON DATA90
Source: J Epidemiol. 2025 Aug 5;35(8):349–54. doi: 10.2188/jea.JE20240298 (PMC12237589; doi:10.2188/jea.JE20240298)
Supplement: Supplementary file 1 [file je-35-349-s001.pdf]

eTable 1. Parameter estimates of logistic regression for estimating transition rates in multistate Markov transition model

Transition			Men			Women		
			Estimates	95% confidence intervals		Estimates	95% confidence intervals	
State 1 →State 2	Intercept		6.5	2.9	10.1	7.0	4.6	9.4
	Age		0.0	-0.1	0.0	-0.1	-0.1	0.0
	Smoking	Ex-smoker	0.2	-0.3	0.8	0.3	-0.4	1.0
		Current	0.1	-0.5	0.6	-0.2	-0.7	0.4
	hypertension	High normal	0.0	-0.9	0.9	0.3	-0.4	0.9
		Grade 1	0.4	-0.4	1.3	0.4	-0.2	1.0
		Grade 2,3	0.5	-0.4	1.4	0.6	-0.1	1.2
	BMI	Underweight	0.3	-0.4	0.9	0.4	-0.1	0.8
		Overweight	0.1	-0.4	0.5	0.1	-0.2	0.4
		Obese	-0.6	-2.6	1.3	0.2	-0.5	0.9
	Diabetes mellitus		-0.1	-0.7	0.5	0.4	0.0	0.8
State 1 →State 3	Intercept		8.3	7.1	9.6	9.9	8.5	11.2
	Age		-0.1	-0.1	-0.1	-0.1	-0.1	-0.1
	Smoking	Ex-smoker	0.1	-0.1	0.4	0.6	0.3	1.0
		Current	0.4	0.2	0.6	0.6	0.4	0.9
	hypertension	High normal	0.1	-0.2	0.4	0.1	-0.3	0.5
		Grade 1	0.2	-0.1	0.5	0.2	-0.1	0.6
		Grade 2,3	0.3	0.0	0.6	0.4	0.0	0.7
	BMI	Underweight	0.2	0.0	0.5	0.4	0.1	0.6
		Overweight	-0.2	-0.4	0.0	0.0	-0.2	0.2
		Obese	0.1	-0.5	0.7	-0.2	-0.7	0.3
	Diabetes mellitus		0.4	0.2	0.6	0.5	0.3	0.8
State 2 →State 1	Intercept		2.3	-60×10e4	60×10e4	48.0	-23×10e4	23×10e4
	Age		0.0	0.0	0.1	0.0	0.0	0.0
	Smoking	Ex-smoker	-1.2	-2.7	0.2	-23.0	-20×10e4	20×10e4
		Current	-0.6	-1.8	0.6	0.9	-0.1	2.0
	hypertension	High normal	22.5	-30×10e4	30×10e4	0.8	-1.2	2.8
		Grade 1	23.1	-30×10e4	30×10e4	0.2	-1.9	2.2
		Grade 2,3	23.0	-30×10e4	30×10e4	0.0	-2.1	2.1
	BMI	Underweight	-23.1	-16×10e4	16×10e4	-23.3	-12×10e4	12×10e4
		Overweight	-0.9	-2.6	0.7	-0.2	-0.9	0.5
		Obese	-22.7	-22.7	-22.7	0.3	-1.1	1.8
	Diabetes mellitus		0.7	-0.9	2.3	0.4	-0.5	1.4
State 2 →State 3	Intercept		6.8	3.4	10.2	9.6	6.2	13.1
	Age		-0.1	-0.1	0.0	-0.1	-0.1	-0.1
	Smoking	Ex-smoker	0.0	-0.5	0.6	0.6	-0.2	1.3
		Current	0.2	-0.4	0.8	0.2	-0.5	0.8
	hypertension	High normal	-0.6	-1.6	0.3	-0.6	-1.3	0.1
		Grade 1	-0.6	-1.5	0.3	-0.7	-1.4	0.1
		Grade 2,3	-0.6	-1.6	0.3	-0.6	-1.3	0.2
	BMI	Underweight	0.3	-0.3	0.9	0.8	0.3	1.4
		Overweight	0.1	-0.5	0.6	0.1	-0.3	0.5
		Obese	0.7	-1.3	2.7	-0.4	-1.4	0.6
	Diabetes mellitus		0.1	-0.6	0.7	0.0	-0.5	0.6

BMI, body mass index.

eTable 2. The Healthy life expectancies at age 65 years according to BP, Smoking status, DM, and BMI using NIPPON DATA 90 (n=6,676; 2,797 men and 3,772 women)

	Non-Communicable Diseases risk factors			Underweight		Normal weight		Overweight		Obese	
	DM	BP	Smoking status		(95%CI)		(95%CI)	HLEs	(95%CI)	HLEs	(95%CI)
**Men**	-	**Normal BP**	**Never-smoker**	20.29	(20.23 — 20.34)	22.62	(22.44 — 22.80)	23.86	(23.69 — 23.86)	21.97	(21.60 — 22.33)
**Men**	-	**Normal BP**	**Ex- smoker**	18.74	(18.61 — 18.88)	20.98	(20.89 — 21.06)	22.10	(22.02 — 22.10)	20.47	(20.19 — 20.74)
**Men**	-	**Normal BP**	**Current smoker**	16.73	(16.69 — 16.78)	18.76	(18.60 — 18.91)	19.87	(19.72 — 19.87)	18.07	(17.75 — 18.40)
**Men**	-	**High normal/Elevated BP**	**Never-smoker**	19.50	(19.29 — 19.71)	22.01	(22.01 — 22.02)	23.09	(23.08 — 23.09)	21.06	(20.87 — 21.25)
**Men**	-	**High normal/Elevated BP**	**Ex- smoker**	18.02	(17.74 — 18.30)	20.27	(20.19 — 20.35)	21.32	(21.22 — 21.32)	19.61	(19.50 — 19.72)
**Men**	-	**High normal/Elevated BP**	**Current smoker**	16.02	(15.84 — 16.20)	18.09	(18.08 — 18.10)	19.12	(19.11 — 19.12)	17.26	(17.09 — 17.43)
**Men**	-	**Grade 1 hypertension**	**Never-smoker**	18.04	(17.89 — 18.20)	20.77	(20.75 — 20.79)	21.57	(21.55 — 21.57)	19.86	(19.60 — 20.11)
**Men**	-	**Grade 1 hypertension**	**Ex- smoker**	16.56	(16.33 — 16.78)	18.88	(18.83 — 18.92)	19.70	(19.65 — 19.70)	18.42	(18.25 — 18.60)
**Men**	-	**Grade 1 hypertension**	**Current smoker**	14.83	(14.70 — 14.95)	16.98	(16.93 — 17.03)	17.83	(17.79 — 17.83)	16.25	(16.02 — 16.48)
**Men**	-	**Grade 2,3 hypertension**	**Never-smoker**	17.17	(16.91 — 17.42)	19.78	(19.73 — 19.83)	20.56	(20.49 — 20.56)	18.92	(18.78 — 19.06)
**Men**	-	**Grade 2,3 hypertension**	**Ex- smoker**	15.73	(15.41 — 16.04)	17.96	(17.83 — 18.09)	18.76	(18.62 — 18.76)	17.53	(17.46 — 17.60)
**Men**	-	**Grade 2,3 hypertension**	**Current smoker**	14.05	(13.83 — 14.26)	16.12	(16.09 — 16.14)	16.95	(16.90 — 16.95)	15.42	(15.29 — 15.55)
**Men**	+	**Normal BP**	**Never-smoker**	17.56	(17.28 — 17.85)	19.63	(19.54 — 19.72)	20.80	(20.68 — 20.80)	18.87	(18.78 — 18.96)
**Men**	+	**Normal BP**	**Ex- smoker**	16.21	(15.87 — 16.55)	18.20	(18.04 — 18.36)	19.28	(19.10 — 19.28)	17.54	(17.52 — 17.56)
**Men**	+	**Normal BP**	**Current smoker**	14.25	(14.00 — 14.49)	16.06	(15.99 — 16.13)	17.12	(17.03 — 17.12)	15.30	(15.21 — 15.38)
**Men**	+	**High normal/Elevated BP**	**Never-smoker**	16.81	(16.39 — 17.23)	19.13	(18.91 — 19.36)	20.14	(19.87 — 20.14)	18.03	(17.97 — 18.09)
**Men**	+	**High normal/Elevated BP**	**Ex- smoker**	15.51	(15.04 — 15.97)	17.56	(17.27 — 17.86)	18.56	(18.23 — 18.56)	16.74	(16.62 — 16.86)
**Men**	+	**High normal/Elevated BP**	**Current smoker**	13.57	(13.21 — 13.93)	15.47	(15.28 — 15.66)	16.44	(16.21 — 16.44)	14.55	(14.50 — 14.60)
**Men**	+	**Grade 1 hypertension**	**Never-smoker**	15.63	(15.28 — 15.98)	18.18	(18.00 — 18.36)	18.98	(18.76 — 18.98)	17.01	(17.00 — 17.02)
**Men**	+	**Grade 1 hypertension**	**Ex- smoker**	14.33	(13.93 — 14.74)	16.48	(16.23 — 16.73)	17.29	(17.02 — 17.29)	15.74	(15.68 — 15.80)
**Men**	+	**Grade 1 hypertension**	**Current smoker**	12.59	(12.30 — 12.89)	14.58	(14.45 — 14.72)	15.41	(15.24 — 15.41)	13.68	(13.67 — 13.70)
**Men**	+	**Grade 2,3 hypertension**	**Never-smoker**	14.82	(14.39 — 15.26)	17.27	(17.04 — 17.51)	18.05	(17.77 — 18.05)	16.16	(16.07 — 16.25)
**Men**	+	**Grade 2,3 hypertension**	**Ex- smoker**	13.57	(13.08 — 14.05)	15.63	(15.32 — 15.94)	16.43	(16.07 — 16.43)	14.93	(14.78 — 15.07)
**Men**	+	**Grade 2,3 hypertension**	**Current smoker**	11.88	(11.51 — 12.25)	13.79	(13.59 — 13.99)	14.60	(14.36 — 14.60)	12.93	(12.86 — 13.00)

BP, blood pressure; CI, confidence interval; DM, diabetes mellitus.

eTable 2. The Healthy life expectancies at age 65 years according to BP, Smoking status, DM, and BMI using NIPPON DATA 90 (n=6,676; 2,797 men and 3,772 women) (continued)

Non-Communicable Diseases risk factors				Underweight		Normal weight		Overweight		Obese	
	DM	BP	Smoking status		(95%CI)		(95%CI)	HLEs	(95%CI)	HLEs	(95%CI)
**Women**	-	**Normal BP**	**Never-smoker**	22.59	(22.52 – 22.65)	26.30	(26.27 – 26.32)	26.11	(26.07 – 26.11)	27.27	(27.25 – 27.28)
**Women**	-	**Normal BP**	**Ex- smoker**	18.15	(18.00 – 18.29)	21.15	(20.96 – 21.33)	21.02	(20.78 – 21.02)	21.81	(21.58 – 22.03)
**Women**	-	**Normal BP**	**Current smoker**	18.79	(18.55 – 19.02)	22.06	(21.83 – 22.29)	22.01	(21.84 – 22.01)	23.20	(22.95 – 23.44)
**Women**	-	**High Normal/Elevated BP**	**Never-smoker**	21.12	(21.11 – 21.13)	25.16	(25.10 – 25.21)	24.87	(24.75 – 24.87)	26.28	(26.27 – 26.30)
**Women**	-	**High Normal/Elevated BP**	**Ex- smoker**	16.89	(16.69 – 17.09)	19.82	(19.57 – 20.07)	19.64	(19.34 – 19.64)	20.34	(20.06 – 20.62)
**Women**	-	**High Normal/Elevated BP**	**Current smoker**	17.62	(17.46 – 17.79)	21.27	(21.09 – 21.44)	21.18	(21.06 – 21.18)	22.53	(22.32 – 22.75)
**Women**	-	**Grade 1 hypertension**	**Never-smoker**	19.98	(19.95 – 20.00)	23.65	(23.64 – 23.65)	23.35	(23.29 – 23.35)	24.49	(24.45 – 24.53)
**Women**	-	**Grade 1 hypertension**	**Ex- smoker**	15.89	(15.73 – 16.05)	18.75	(18.54 – 18.95)	18.56	(18.30 – 18.56)	19.22	(18.98 – 19.45)
**Women**	-	**Grade 1 hypertension**	**Current smoker**	16.63	(16.44 – 16.83)	19.98	(19.77 – 20.20)	19.87	(19.71 – 19.87)	21.09	(20.82 – 21.36)
**Women**	-	**Grade 2,3 hypertension**	**Never-smoker**	18.93	(18.87 – 18.99)	22.44	(22.42 – 22.47)	22.14	(22.11 – 22.14)	23.17	(23.12 – 23.21)
**Women**	-	**Grade 2,3 hypertension**	**Ex- smoker**	14.98	(14.84 – 15.11)	17.76	(17.59 – 17.93)	17.56	(17.34 – 17.56)	18.18	(17.98 – 18.38)
**Women**	-	**Grade 2,3 hypertension**	**Current smoker**	15.73	(15.51 – 15.95)	18.95	(18.72 – 19.18)	18.83	(18.65 – 18.83)	19.98	(19.71 – 20.25)
**Women**	+	**Normal BP**	**Never-smoker**	18.31	(18.24 – 18.39)	21.65	(21.59 – 21.71)	21.47	(21.47 – 21.47)	22.54	(22.46 – 22.62)
**Women**	+	**Normal BP**	**Ex- smoker**	14.35	(14.24 – 14.46)	17.03	(16.88 – 17.18)	16.92	(16.73 – 16.92)	17.62	(17.45 – 17.80)
**Women**	+	**Normal BP**	**Current smoker**	14.91	(14.68 – 15.14)	17.95	(17.70 – 18.20)	17.91	(17.72 – 17.91)	19.05	(18.76 – 19.33)
**Women**	+	**High Normal/Elevated BP**	**Never-smoker**	17.01	(17.00 – 17.02)	20.82	(20.80 – 20.83)	20.56	(20.52 – 20.56)	21.91	(21.82 – 21.99)
**Women**	+	**High Normal/Elevated BP**	**Ex- smoker**	13.24	(13.08 – 13.40)	15.85	(15.65 – 16.05)	15.70	(15.45 – 15.70)	16.32	(16.09 – 16.55)
**Women**	+	**High Normal/Elevated BP**	**Current smoker**	13.87	(13.71 – 14.04)	17.31	(17.10 – 17.52)	17.26	(17.09 – 17.26)	18.50	(18.25 – 18.74)
**Women**	+	**Grade 1 hypertension**	**Never-smoker**	16.00	(15.95 – 16.04)	19.42	(19.36 – 19.48)	19.16	(19.15 – 19.16)	20.27	(20.14 – 20.39)
**Women**	+	**Grade 1 hypertension**	**Ex- smoker**	12.36	(12.24 – 12.49)	14.89	(14.73 – 15.06)	14.73	(14.52 – 14.73)	15.32	(15.13 – 15.51)
**Women**	+	**Grade 1 hypertension**	**Current smoker**	13.00	(12.81 – 13.19)	16.13	(15.88 – 16.39)	16.05	(15.84 – 16.05)	17.21	(16.90 – 17.52)
**Women**	+	**Grade 2,3 hypertension**	**Never-smoker**	15.07	(14.99 – 15.14)	18.34	(18.27 – 18.41)	18.07	(18.05 – 18.07)	19.08	(18.96 – 19.21)
**Women**	+	**Grade 2,3 hypertension**	**Ex- smoker**	11.57	(11.47 – 11.66)	14.01	(13.88 – 14.15)	13.85	(13.67 – 13.85)	14.40	(14.25 – 14.56)
**Women**	+	**Grade 2,3 hypertension**	**Current smoker**	12.20	(12.00 – 12.41)	15.20	(14.94 – 15.46)	15.10	(14.89 – 15.10)	16.21	(15.89 – 16.53)

BMI, body mass index; BP, blood pressure; CI, confidence interval; DM, diabetes mellitus; HLE, healthy life expectancy.
Underweight: BMI <18.5 kg/m^2; Normal weight: BMI 18.5–24.9 kg/m^2; Overweight: BMI 25.0–29.9 kg/m^2; Obese: BMI ≥30.0 kg/m^2.
Diabetes mellitus (+) was defined as a history of diabetes mellitus, Hb1Ac ≥ 6.5%, and/or use of antidiabetic medications.
Normal BP: systolic BP <120 mm Hg and diastolic BP <80 mm Hg; High normal/elevated BP: systolic BP 120–139 mm Hg and/or diastolic BP 80–89 mm Hg; Grade I hypertension: systolic BP 140–159 mm Hg and/or diastolic BP 90–99 mm Hg; Grade II/III hypertension: systolic BP ≥160 mm Hg and/or diastolic BP ≥100 mm Hg. Smoking status was categorized into three groups (never-smokers, ex-smokers, and current smokers).

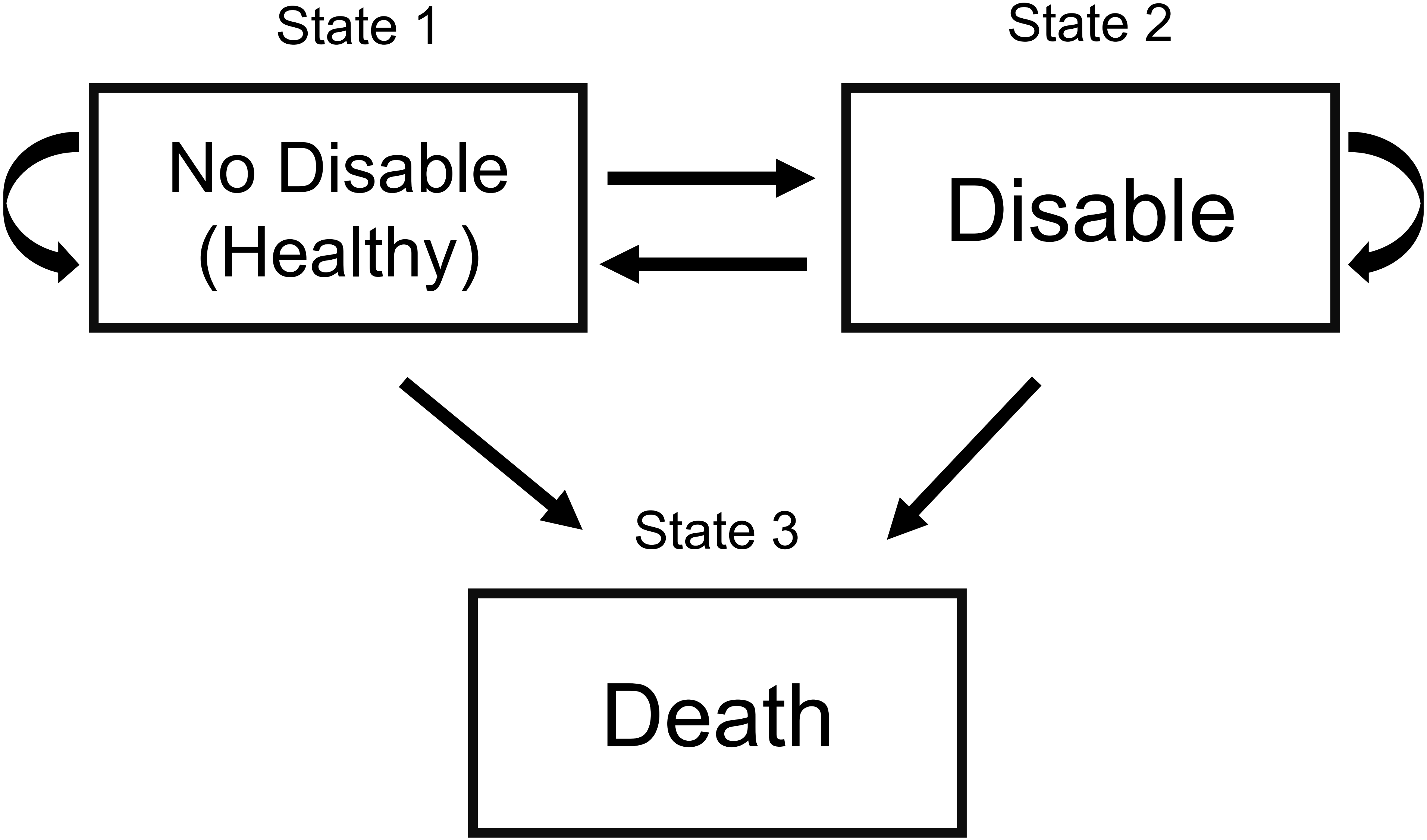

eFigure 1. Multistate Markov model used with individuals being in one of three possible states at time.
